# The association between continuous metabolic syndrome score and its components with electrocardiographic abnormalities in community-dwelling older adults: the Bushehr elderly health (BEH) program

**DOI:** 10.1186/s12872-024-03733-1

**Published:** 2024-01-31

**Authors:** Akram Farhadi, Hadi Emamat, Reza Nemati, Maryam Marzban, Gita Shafiee, Iraj Nabipour, Afshin Ostovar, Zahrasadat Jalaliyan, Hasan Malekizadeh, Bagher Larijani

**Affiliations:** 1 The Persian Gulf Tropical Medicine Research Center, The Persian Gulf Biomedical Sciences Research Institute, Bushehr University of Medical Sciences, Bushehr, Iran; 2 Department of Medical Emergencies, School of Allied Medical Sciences, Bushehr University of Medical Sciences, Bushehr, Iran; 3 Clinical Research Development Center, The Persian Gulf Martyrs Hospital, Bushehr University of Medical Sciences, Bushehr, Iran; 4 Department of Biostatistics and Epidemiology, Faculty of Health and Nutrition, Bushehr University of Medical Sciences, Bushehr, Iran; 5 Statistical Genetics Lab, QIMR Berghofer Medical Research Institute, Brisbane, QLD Australia; 6 Chronic Diseases Research Center, Endocrinology and Metabolism Population Sciences Institute, Tehran University of Medical Sciences, Tehran, Iran; 7 The Persian Gulf Marine Biotechnology Research Center, The Persian Gulf Biomedical Sciences Research Institute, Bushehr University of Medical Sciences, Bushehr, Iran; 8 Osteoporosis Research Center, Endocrinology and Metabolism Clinical Sciences Institute, Tehran University of Medical Sciences, Tehran, Iran; 9 School of Medicine, Bushehr University of Medical Sciences, Bushehr, Iran; 10 Endocrinology and Metabolism Research Center, Endocrinology and Metabolism Clinical Sciences Institute, Tehran University of Medical Sciences, Tehran, Iran

**Keywords:** Metabolic syndrome, Aged, Electrocardiography, Cardiovascular disease

## Abstract

**Background:**

Metabolic syndrome (MetS) known as a risk factor for cardiovascular diseases (CVDs) has developed into a major source of health issue, especially for the elderly. In the present study, we investigated the association between continuous MetS (cMetS) score and its components with electrocardiographic (ECG) abnormalities in the community-dwelling older adults.

**Methods:**

This cross-sectional study is derived from the second phase of BEH cohort study which is conducted on individuals aged over 60 years old. Standard 12-lead ECGs were recorded and coded by qualified physicians and continuous values of metabolic syndrome risk scores (cMetS) were measured. Data regarding socio-demographic, medical history, and lifestyle variables were collected by trained interviewers. The multinomial regression analysis was used to investigate the relationship between cMetS and its components with ECG abnormalities in the included participants.

**Results:**

2426 individuals (mean age ± standard deviation: 69.30 ± 6.33 years) were included in the final analysis. Overall, 22.5% of the participants showed ECG abnormalities. Among these, 8.0% (*n* = 139) of participants had minor and 14.6% (*n* = 354) had major ECG abnormalities. In the final models, cMetS (OR = 1.04), mean arterial pressure (MAP((OR = 1.01), and higher fasting blood glucose (FBG) (OR = 1.01) increased the risk of ECG abnormalities (*p* < 0.05). Also, cMetS (OR = 1.05) and MAP (OR = 1.02) were associated with an increased risk of major ECG abnormalities (*p* < 0.05).

**Conclusion:**

MetS and MAP were significantly associated with ECG abnormalities. The results of the present study suggest that ECG screening in the older population with MetS could potentially help to detect those at the higher risk of CVDs.

**Supplementary Information:**

The online version contains supplementary material available at 10.1186/s12872-024-03733-1.

## Background

Metabolic syndrome (MetS), a term used to describe a series of metabolic anomalies including abdominal obesity, increased blood pressure, disturbed glucose homeostasis, and dyslipidemia, has been associated with increased risk of cardiovascular diseases (CVDs) and mortality [1]. The prevalence of metabolic syndrome varies from 12.5 to 31.4% in different parts of the world. In the Eastern Mediterranean region and the Americas, the prevalence of metabolic syndrome was higher than in other parts of the world [2]. The prevalence of metabolic syndrome in Iran varies from 32 to 47.6% based on different criteria [3]. These statistics show the high prevalence of this syndrome in Iran and the world.

The electrocardiogram (ECG) has been traditionally used as a simple and practical tool to diagnose and predict coronary heart disease (CHD) and other CVDs [4]. The study of Yazdanpanah et al. (2020) showed that the parameters of P duration, PR interval, QTc interval, and QRS duration were higher in people with metabolic syndrome, and the parameters of P amplitude II, R amplitude V5, P axis, and QRS axis were lower than healthy people [5]. The results of the Elffers study in 2017 were also somewhat similar to the Yazdanpanah study [6]. Continuous metabolic syndrome (cMetS) score has been suggested as a tool of both high sensitivity and high specificity to ascertain the existence of MetS [7].

However, there are contradictions in the current studies regarding the relationship between ECG abnormalities and MetS; various covariates, including gender, genetics, race, environmental, and lifestyle factors might be responsible for these heterogeneities. As an example, for such inconsistencies, one study showed that there is a significant relationship between certain components of the MetS and ECG abnormalities only in men [8]. Conversely, other studies have reported that MetS and its components, especially waist circumference and blood pressure, were associated with ECG abnormalities in the overall population [5, 6]. On the other hand, Richard et al. (2023) reported that ECG abnormalities in women, compared to men, had a higher likelihood of developing metabolic syndrome [9]. Soflaei et al. (2023) reported that an increase in high-density lipoprotein cholesterol was negatively associated with ischemic ECG manifestations, while a significant positive association was observed only with an ST segment elevation [10]. However, in another study, the prevalence of high-density lipoprotein cholesterol was higher in patients with acute coronary syndrome [11].

The number of the elderly in the community is increasing rapidly, as it is the prevalence of MetS; moreover, there is a growing need to find and validate tools for the early prediction of CVDs. On the other hand, the present literature shows a high level of inconsistency. Also, using cMetS instead of binary definition have some advantages in epidemiologic studies. Increasing statistical power, more sensitive and less error-prone are benefits of cMetS compared to dichotomizing MetS. Additionally, the use of cMetS index is another vacancy in the knowledge regarding the possible relationship between ECG abnormalities and MetS. Therefore, the aim of the present study is to determine the association between cMetS and its components with electrocardiographic abnormalities in in community-dwelling older adults.

## Materials and methods

### Research design and participants

This cross-sectional study was conducted based on the data of the second phase of Bushehr Elderly Health (BEH) program. The BEH program is a population-based prospective cohort study to investigate the risk factors for non-communicable diseases (NCDs) in a sample of older adults aged ≥ 60 years in the urban population of Bushehr, Iran in North of the Persian Gulf. The study design and methodology has been published elsewhere [12, 13]. Based on the classifications made by the municipality, we divided the city of Bushehr into 75 regions and the participants were selected using a multi-stage cluster random sampling method, in which each region was assigned a number and then the numbers were arranged randomly. All eligible elderly in each region were invited to the study, and in some cases, the invitation process was repeated several times to reach the required sample size for that group. Inclusion criteria in the BEH study were: both sexes of people with age more than or equal to 60 years that were residents in Bushehr port since at least 1 year before the recruitment and did not want to leave Bushehr for the following 2 years after the recruitment. Also, people with adequate physical and mental ability participated in this program. People who did not live in Bushehr and were unwilling to participate were excluded from the study. Initially, 3297 individuals were screened of which 3000 were eligible to participate in the first phase of the study, yielding a participation rate of 91%. The first and second phases were completed in 2013–2014 and 2015–2018 time periods, respectively. The participation rate in the second phase was 81%.

### Measurement

Demographic information form, medical records and factors related to lifestyle were completed during an interview.

A fixed stadiometer was used to measure height. This instrument, should be placed vertically on a rigid horizontal surface without movement, so we placed its base on the floor of the room, with no carpets or any soft materials. The floor of the room should be hard (mosaic, cement, etc.) and should not be carpeted or covered with other soft materials. We asked the person to take off his shoes and heavy clothes (coat, jacket, etc.), if any. To measure height, a person must stand straight so that the back of the head, shoulders, hips, legs and heels are tangent to the vertical surface of height measurement. The heels should also be somewhat close to each other. We asked the person to look straight ahead so that the external ear hole is along the lower edge of the eye socket. Then, the horizontal plate on the height measuring tool was slowly lowered until it placed on the person’s head so that the hair was compressed.

Women’s height was measured without head covering (hijab, scarf, or hat). These measurements were made by a female examiner. Height was recorded in centimeters. It is not acceptable to record the height based on the person’s statement for normal people. Only in people who are unable to move (legs amputated, sitting in a wheelchair, etc.) the height is recorded as they report it.

A digital scale was used to measure the weight, which was placed on a hard and unfurnished surface. The display must show zero before measuring. The person took off the shoes and also the heavy clothes and went on the scale, standing in the center of the plate. Improper standing may cause inaccurate weight measurement. The weight was recorded in the form when the digital number reached a balance mode. The weight that the person reports is only acceptable for people who are not able to move (such as amputated legs, etc.). The person who has stepped on the scale should never try to read the weight himself. If the digital scale was moved, the device was calibrated with a standard weight.

Body mass index (BMI) was determined by dividing the weight in kg by the square of height in meters (Kg/m^2^).

The tool used to measure waist circumference (WC) was a tape measure. The person stood straight and looked ahead. The tape measure was tied at the narrowest point of the waist, in the navel area. The person was breathing normally and calmly and did not hold his breath in his chest. The tape must be completely horizontal on both the front and back sides. For this purpose, a mirror was used to ensure the correct placement of the tape measure. It should not be closed so tightly that it compresses the skin and tissues. The corresponding number is recorded in centimeters.

Current smoker was defined as one who smokes at least one cigarette per day or uses a hookah or pipe once daily at the time of evaluation. The amount of physical activity was estimated based on metabolic equivalents (METs) using a validated questionnaire for a single measurement of 24 hours’ physical activity on an average weekday [14].

After 15 min of rest in a seated position, the blood pressure (BP) was measured on the right arm using a standardized mercury sphygmomanometer. Following an interval of five minutes, two consecutive BP measurements were made and the averages of systolic and diastolic pressure were reported in mmHg. Mean arterial pressure (MAP) was calculated using the following formula: MAP = [(SBP–DBP)/3] + DBP.

Venous blood samples were collected from participants following 8–12 of fasting. The biochemical parameters were measured via laboratory testing in a fasting state, according to the standard protocols. Serum lipid profiles and fasting plasma glucose (FPG) were measured by an enzymatic colorimetric technique using a commercial kit (Pars Azmun, Karaj, Iran).

### Electrocardiogram

A resting 12-lead electrocardiograms (ECGs) were obtained from participants in a supine position at 10 mm/mV calibration and speed of 25 mm/s according to the standard recording protocol. Two qualified physicians coded the ECGs simultaneously using the Minnesota codes with a measuring loupe specially manufactured by University of Minnesota [15]. To resolve their disagreement, a third qualified physician, re-evaluated and finally approved the ECG codes. Then, for assurance of quality, a cardiologist recoded 10% of ECGs. The Minnesota Code (MC) is a classification system for the ECG to investigate the association between the ECG abnormalities and cardiovascular risk factors.

The following categories were considered in coding: Q-QS pattern (1-Codes), QRS Axis deviation (2-Codes), High Amplitude R Waves (3-Codes), STsegment depression (4-Codes), Twave pattern (5-Codes), atrioventricular (AV) conduction defects (6-Codes), ventricular conduction defects (7-Codes).

X), arrhythmias (8-Codes) and Miscellaneous Codes (9-Codes). ECGs were classified minor abnormalities, major abnormalities and normal according to change of duration and/or voltage of the Q wave, ST-segment/T-wave abnormalities, and prolonged QRS. The details of the Minnesota Code Classification System for Electrocardiographic Findings are shown in Supplementary 1.

Therefore, major ECG abnormalities were defined as manifestation of any of the following: Q-QS wave abnormalities (MC 1–1 to 1-2-8); left ventricular hypertrophy (MC 3 − 1); Wolff–Parkinson–White syndrome (MC 6-4-1 or 6-4-2); complete bundle branch block or intraventricular block (MC 7-1-1, 7-2-1, 7 − 4, or 7–8); atrial fibrillation or atrial flutter (MC 8 − 3); or major ST-T changes (MC 4 − 1, 4 − 2, 5 − 1, and 5 − 2). Minor ECG abnormalities were defined as showing minor ST-T changes (MC 4 − 3, 4–4, 5 − 3, and 5 − 4). Participants with both major and minor abnormalities were classified as having major abnormalities. Participants without minor or major ECG abnormalities were classified as having no abnormalities and their ECG was considered normal [15, 16].

### Continuous metabolic syndrome definition

cMetS score was computed by the use of standardized residuals (Z-scores) of variables (WC, MAP, HDL-c, TG, and FBG). Computation of Z-scores for the variables was performed by regressing each factor on age, and sex to account for age- and sex-related differences. Then, the standardized residual of variables were saved (e.g., Z_WC, etc.) [17]. Given that the standardized HDL-c and MetS risk are inversely correlated, it was multiplied by -1. The standardized residuals (z scores) for each of the individual variables were added to determine the cMetS score. The same variables were utilized in adult clinical MetS criteria. MAP was used since including systolic and diastolic blood pressure would load 2 blood pressure variables in its calculation A higher cMetS score indicates a less favorable MetS profile [18].

### Ethical approval

The Research Ethics Committee of Bushehr and Tehran University of Medical Sciences approved the protocol of the BEH program (Ethical Code: IR.BPUMS.REC.1401.067). Also, written informed consents were obtained from all participants. Informed consent has also been obtained for all illiterate participants from their parents and/or legal guardians.

### Statistical analysis

The data are presented as mean ± standard deviation (SD) for continuous and as percentage for qualitative variables. Variables were also compared across categories of ECG abnormality using the ANOVA test for continuous variables and the chi-squares test for qualitative variables.

Age, sex, education, current smoking, physical activity, high fat mass, daily diet intakes and BMI, were considered as the main set of variables. The Akaike Information Criterion (AIC) (the best present method) was used to select the final model from all possible subsets. The multinomial regression analyses were used to investigate the associations of cMetS and its components across ECG abnormality categories. Results were presented as odds ratios (ORs) and 95% confidence intervals. Data were analyzed using the Stata 14 software (StataCorp. 2015, Stata Statistical Software: Release 14. College Station, TX: StataCorp LP) and *p*-values ≤ 0.05 were considered as statistically significant.

## Results

A total of 2426 individuals were found eligible to enroll in the study (51.65% females) with the mean age of 69.30 ± 6.33. Baseline characteristics of the study participants across ECG categories are presented in Table 1. A fifth of the participants 547 (22.5%) had ECG abnormalities among whom 193 (7.9%) and 354 (14.6%) had minor ECG and major ECG abnormalities, respectively. Subjects in the group with major ECG abnormalities were older (70.12 ± 6.60 vs. 69.14 ± 6.32), had fewer years of education (4.64 vs. 5.37), and higher mean of MAP (102.87 ± 11.95 vs. 100.61 ± 10.53) than subjects with normal ECG (*p*-value < 0.05).

**Table 1:** General characteristics of the study population according to ECG status

	ECG Category			
	Normal (No ECG abnormality) (*n* = 1879)	Minor ECG abnormality (*n* = 193)	Major ECG abnormality (*n* = 354)	*P*-value
Sex (Men), %	939(50.0)	50(25.9)	177(50.0)	0.104
Physical activity, %	439(23.4)	46(23.8)	70(19.8)	0.186
Current Smoking, %	387(20.6)	39(20.2)	78(22.1)	0.584
Age, (Years)	69.14 ± 6.32	69.95 ± 6.69	70.12 ± 6.60	0.012
Waist circumference (cm)	98.67 ± 11.76	100.09 ± 12.01	98.20 ± 13.31	0.200
Hip circumference (cm)	102.51 ± 10.02	103.98 ± 10.80	102.11 ± 10.53	0.104
BMI, (Kg/m^2^)	27.50 ± 4.90	27.92 ± 5.22	27.37 ± 4.73	0.436
Education, (Years)	5.37 ± 5.05	4.58 ± 4.87	4.64 ± 4.92	0.008
MAP	100.61 ± 10.53	100.45 ± 11.11	102.87 ± 11.95	0.001
FBG (mg/dl)	105.48 ± 41.07	109.74 ± 50.31	108.28 ± 45.80	0.258
TG (mg/dl)	136.18 ± 71.65	135.18 ± 64.97	134.97 ± 66.90	0.946
HDL-C (mg/dl)	45.94 ± 11.06	47.28 ± 12.29	45.21 ± 11.40	0.120
cMetS score	-0.06 ± 2.76	0.09 ± 3.00	0.27 ± 2.88	0.110

Data are presented as n (%) or mean ± standard deviation

BMI; Body Mass Index, FBG; fasting blood glucose, TG; triglyceride, HDL-c; high-density lipoprotein cholesterol, MAP; mean arterial pressure, cMetS; continuous metabolic syndrome

Table 2 presents the results of the multinomial logistic regression models to examine the association between cMetS and its components and minor and major ECG abnormalities. cMetS was associated with an increased risk of major ECG abnormalities in crude model (OR:1.04(95% CI : 1.01–1.09). After adjustment for confounders (age, sex, smoking, physical activity, education and body mass index) in the final model, the risk for major ECG abnormality remained significant (OR = 1.05, 95% CI: 1.01–1.10). Moreover, MAP was positively and significantly associated with major ECG abnormalities in crude and adjusted models (OR = 1.02, 95% CI: 1.01–1.03). However, waist circumference was significantly associated with minor ECG abnormality in full adjusted model, this association was disappeared with major ECG abnormalities.

**Table 2:** Association of cMetS and its components with minor and major ECG abnormalities

	No ECG abnormality	Minor ECG abnormality	Major ECG abnormality
	OR (95% CI)	OR (95% CI)	OR (95% CI)
cMetS			
Crude Model	1.00	1.02(0.97–1.08)	**1.04(1.01–1.09)**
Model 1	1.00	1.02(0.97–1.08)	**1.04(1.00-1.09)**
Model 2	1.00	1.03(0.97–1.10)	**1.05(1.01–1.10)**
MAP			
Crude Model	1.00	0.99(0.98–1.01)	**1.02(1.01–1.03)**
Model 1	1.00	1.00(0.98–1.02)	**1.02(1.01–1.03)**
Model 2	1.00	1.00(0.98–1.02)	**1.02(1.01–1.03)**
TG			
Crude Model	1.00	0.99(0.99-1.00)	0.99(0.99-1.00)
Model 1	1.00	0.99(0.99-1.00)	0.99(0.99-1.00)
Model 2	1.00	0.99(0.99-1.00)	0.99(0.99-1.00)
Waist circumference			
Crude Model	1.00	1.01(0.99–1.02)	0.99(0.98–1.01)
Model 1	1.00	1.01(0.99–1.02)	0.99(0.98–1.01)
Model 2	1.00	**1.02(1.00-1.05)**	0.99(0.98–1.01)
HDL-C			
Crude Model	1.00	1.01(0.99–1.02)	0.99(0.98-1.00)
Model 1	1.00	0.99(0.98–1.01)	0.99(0.98-1.00)
Model 2	1.00	0.99(0.98–1.01)	0.99(0.98-1.00)
FBG			
Crude Model	1.00	1.00(0.99–1.01)	1.00(0.99-1.00)
Model 1	1.00	1.00(0.99–1.01)	1.00(0.99-1.00)
Model 2	1.00	1.00(0.99–1.01)	1.00(0.99-1.00)

Data are presented as odds ratios (ORs) and 95% confidence intervals (95% CIs). FBG: fasting blood glucose, HDL-c: high-density lipoprotein cholesterol, MAP: mean arterial pressure, cMetS: continuous metabolic syndrome

Model 1: adjusted for age and sex

Model 2: adjusted for age, sex, smoking, physical activity, education and body mass index

Table 3 shows the association between cMetS and its components and the existence of any ECG abnormalities. cMets was associated with any ECG abnormalities in crude model and remained unchanged in adjusted models (OR = 1.04, 95% CI:1.01–1.08). Also, MAP was associated with an increased risk of any ECG abnormalities in all models (OR_adj_=1.01, 95% CI:1.00-1.02). The association of FBG was weak for any ECG abnormalities. Contrarily, other components of cMetS did not show significant relationships with any of the ECG abnormalities.

**Table 3:** Association of cMetS and its components with any ECG abnormalities

	No ECG abnormality	Any ECG abnormality
cMetS		
Crude Model	1.00	**1.04(1.00-1.07)**
Model 1	1.00	**1.04(1.01–1.07)**
Model 2	1.00	**1.04(1.01–1.08)**
MAP		
Crude Model	1.00	**1.01(1.00-1.02)**
Model 1	1.00	**1.01(1.00-1.02)**
Model 2	1.00	**1.01(1.00-1.02)**
TG		
Crude Model	1.00	1.00(0.99-1.00)
Model 1	1.00	1.00(0.99-1.00)
Model 2	1.00	1.00(0.99-1.00)
Waist circumference		
Crude Model	1.00	1.00(0.99–1.01)
Model 1	1.00	1.00(0.99–1.01)
Model 2	1.00	1.00(0.99–1.02)
HDL-C		
Crude Model	1.00	1.00(0.99–1.01)
Model 1	1.00	0.99(0.98-1.00)
Model 2	1.00	0.99(0.98–1.01)
FBG		
Crude Model	1.00	**1.01(1.00-1.01)**
Model 1	1.00	**1.00(1.00-1.01)**
Model 2	1.00	**1.00(1.00-1.01)**

Data are presented as odds ratios (ORs) and 95% confidence intervals (95% CIs). FBG: fasting blood glucose, HDL-C: high-density lipoprotein cholesterol, MAP: mean arterial pressure, cMetS: continuous metabolic syndrome

Model 1: adjusted for age and sex

Model 2: Additionally, adjusted for smoking, physical activity, education and, body mass index.

## Discussion

The present study is the first to investigate the association between cMetS and its components with ECG abnormalities in community-dwelling older adults of Bushehr (southern Iran). The results of this study indicated that cMetS and MAP, as one of its important components, are associated with the existence of any ECG abnormalities. Additionally, cMetS and MAP were significantly associated with the presence of major ECG abnormalities.

Previous studies consider various variables to be effective on the risk of cardiovascular diseases. According to the latest report of the National Institute of Health Research of Iran in 2016, the people of Bushehr province were introduced as having the least amount of physical activity among the provinces of Iran [19]. It is widely accepted that sedentary lifestyle is associated with the development of MetS [20] and MetS, in turn, could augment the risk of various CVDs [21]. Also, smoking, especially water pipe smoking, is relatively common in Bushehr city [22], and it is accepted that smoking acts as one of the main risk factors for cardiovascular diseases [23]. Therefore, we adjusted physical activity and smoking variables in the models.

In the present study, the prevalence of ECG abnormalities in the elderly was 22.5%. Furthermore, the frequency of minor and/or major ECG abnormalities was significantly higher in older subjects with lower education levels and higher blood pressures. This observation is also consistent with the existing literature which shows that ECG abnormalities are more common in the elderly and that there is a linear positive correlation between age and such anomalies, especially in the range of 30–70 years of age [24, 25]. The inverse relationship between education level and the risk of CVDs has also been previously discussed by the American Heart Association (AHA) [26]. Additionally, there is a general consensus regarding the association between higher blood pressure and the risk of CVDs and the significance of its management in the prevention and treatment of heart problems [27]. These studies show that our findings can confirm the significance of abnormal ECGs and high blood pressure in the etiology, hence the prevention of CVDs.

Studies have shown that obesity and increased body fat mass are associated with the increased risk of ECG abnormalities [28]; moreover, it has been shown that central (abdominal) obesity plays a much crucial role in the mentioned association [29, 30]. Although the overall association between body fat mass and CVDs is rarely contested, the literature regarding the older adults has been heterogeneous [31]. There is some evidence suggesting that the association between body fat mass and CVDs is diluted with the increasing age [32]. Such findings might partly justify our inability to detect such a relationship, for our target population was the elderly. Surprisingly, in a study on the seniors, Ohori et al. even found that a high percentage of body fat mass might function as a possible protection against CVDs and cardiac events. They proposed that since heart failure (HF) can cause anorexia and cachexia and that the adipose tissue (AT) can provide energy through mobilization and catabolism of its sources of fatty acids, increased mass of AT might be preventive against other cardiac events and mortality, at least in the advanced stages of HF [33].

We were also able to show that the MetS is associated with the existence of any ECG abnormalities. In the light of the fact that ECG abnormalities positively correlate with the risk of CVDs [34, 35], our findings reconfirm the significance of MetS as a valid predictor of morbidity and mortality from these ailments [36–38]. Our results also showed that among the components of MetS, MAP is associated with the presence of any ECG abnormalities. The relationship between hyperglycemia and elevated blood pressure and CVDs is well-established [27, 39–41]. In the long-term, hypertension causes left atrial and ventricular remodeling; the subsequent left ventricular hypertrophy (LVH) can cause distortions in ECGs of these patients [42]. Accordingly, LVH has been traditionally considered as a cornerstone of risk assessment of cardiovascular events in hypertensive subjects [43]. Likewise, abundant evidence exists indicating that hyperglycemia plays an unignorably role in favor of infliction with CVDs. Several mechanisms have been proposed, including the microvascular damage caused by exposure to long-term elevated glucose levels [44, 45]. As a matter of fact, some disturbances in ECG, such as sinus tachycardia, long QTc, QT dispersion, changes in heart rate variability, ST-T changes, and LVH have been reported in early stages of diabetes mellitus [46].

We did not find a significant association between increased TG and reduced HDL-c levels and the presence of ECG abnormalities. Ebong et al. reported that the association between increased TG levels and ECG abnormalities was only significant in females; they also failed to observe any associations between lowered HDL-c levels and ECG anomalies [47]. Another study suggested that the relationship between ECG parameters and a disturbed lipid profile might be altered based on the gender [5]. However, in the latter study, the researchers addressed various ECG parameters as their major outcome, while we focused more on the known ECG anomalies. In contrast, another study indicated that higher TG to HDL-c ratio (rather than each separately) might exert a synergistic effect with ischemic ECG changes on fatal ischemic heart disease (IHD) [26]. In summary, we can deduce that the knowledge regarding the effect of increased TG and decreased HDL-c levels on ECG abnormalities is widely limited and incongruous; plus, the exact underlying mechanisms have not been yet elucidated. Hence, all components of Mets do not increase the risk of ECG abnormalities, it seems that individual components may act synergistically1 to result in ECG abnormalities. Previous studies showed that the increasing metabolic syndrome score provided a graded assessment of CVD risk [48, 49].

### Clinical implications

This study suggests that routine electrocardiogram (ECG) screening for individuals with cMetS, particularly in elderly subjects, may aid in early detection of cardiovascular disease (CVD) symptoms, effectively identifying high-risk cases to prevent severe complications. As cardiac MetS (cMetS) is linked to increased risks of cardiovascular complications and mortality, the use of ECG as an early prognostic tool can be crucial in preventing further health issues. Integrating ECG assessments into routine health check-ups of the elderly with MetS is paramount, urging healthcare policymakers and managers to plan strategies for reducing the burden of cardiovascular-related diseases and Metabolic Syndrome. Continuous training programs for healthcare staff on metabolic abnormalities and ECG should be implemented, empowering staff to effectively manage these health conditions. Clinical nurses, prompted by the study’s findings, can provide specialized attention to elderly individuals diagnosed with MetS, focusing on particular ECG abnormalities to potentially prevent heart-associated complications.

### Strengths, limitations, and suggestions

To the best of our knowledge, this is the first large population-based study to examine the association between MetS and ECG abnormalities as a subclinical index of heart disease in the elderly population. Additionally, in this study we took advantage of the cMetS score which is a more valid indicator of MetS than the categorical (having/not having) criteria customarily used [50]. However, this study surely has got some limitations. First, due to the cross-sectional nature of the study, a causal interpretation of our findings is rendered impossible. Another limitation of this study is that there existed a vacancy concerning the use of medications and assessment of individual nutrition of the participants which may act as confounding covariates in the fitted models. Also, using a physical activity questionnaire to measure physical activity was one of the limitations of this study. Also, this research has been conducted on individuals from a city in southern Iran. Therefore, the generalizability of the results to other cultures, ethnicities, and races should be approached with caution.

It is recommended to conduct longitudinal future studies to determine causal relationships between cMetS, ECG abnormalities, and cardiovascular complications. Long-term observational studies can confirm findings and enhance the understanding of these relationships. Researchers in future studies should incorporate an assessment of medications and individual nutrition among participants. These variables might act as confounding factors influencing findings and need to be considered for a more comprehensive understanding of their role in the association between cMetS and ECG abnormalities. Additionally, it is suggested that by exploring alternative methods, a more accurate measurement of physical activity should be performed. Utilizing a better approach, such as precise tools for measuring physical activity, can provide more accurate and comprehensive data regarding participants’ physical activity levels. In future studies, it is proposed that an intervention study designs extensive educational programs for patients and healthcare providers focusing on MetS, ECG abnormalities, and the management of these conditions.

## Conclusion

Our results indicated that cMetS and MAP, as an important component of MetS, are associated with the presence of any kind of ECG abnormalities. Since cMetS is associated with an increased risk of cardiovascular complications and mortality, and as ECG abnormalities are used as prognostic indices to discover heart-associated problems, the use of ECG in subjects with cMetS may be condoned for earlier and better identification of high-risk cases of heart disease. Therefore, we propose that routine ECG screening might be recommended in elderly subjects with MetS to detect symptoms of CVDs as early as possible and, thus, to avoid the following severe complications.

These conclusion and results highlight the importance of the attention of managers and policy makers of the health system to the use of electrocardiography in the elderly. It also helps managers in the field of medical education to plan and develop training programs in the form of continuous training for treatment staff; In these programs, participants’ knowledge about MetS and ECG and how to deal with these problems in patients should be reminded. The findings of the present study help clinical nurses to pay special attention to the most important criteria and abnormalities in the ECG in the elderly with MetS.

### Electronic supplementary material

Below is the link to the electronic supplementary material.

Supplementary Material 1: Classifications used by the Minnesota Code for ECG abnormality

## Data Availability

The datasets used and/or analyzed during the current study are available from the corresponding author on reasonable request.

## Abbreviations

BEH: Bushehr elderly health
cMetS: Continuous
AHA: American Heart Association; Measurements of Metabolic Syndrome
SBP: Systolic blood pressure
DBP: Diastolic blood pressure
BMI: Body mass index
ECG: Electrocardiographic
MAP: Mean arterial pressure
LVH: Left ventricular hypertrophy
WHR: Waist to hip ratio
HTN: Hypertension
HDL: High-density lipoproteins
LDL: Low-density lipoproteins
TG: Triglycerides
Hgb: Hemoglobin
RBC: Red blood cells
HbA1c: Hemoglobin A1c
ECG: Electrocardiogram
