# Supplementary material for: The association between continuous metabolic syndrome score and its components with electrocardiographic abnormalities in community-dwelling older adults: the Bushehr elderly health (BEH) program
Source: BMC Cardiovasc Disord. 2024 Jan 31;24:81. doi: 10.1186/s12872-024-03733-1 (PMC10829232; doi:10.1186/s12872-024-03733-1)
Supplement: Supplementary file 1 — Supplementary Material 1: Classifications used by the Minnesota Code for ECG abnormality [file 12872_2024_3733_MOESM1_ESM.docx]

**Supplementary 1: Classifications used by the Minnesota Code for ECG abnormality**

| **Classifications used by the Minnesota Code for ECG abnormality** | |
| --- | --- |
| 1-1-1 . . . . . .1-3-6 | Q waves |
| 2-1 . . . . . .2-5 | QRS axis deviation |
| 3-1 . . . . . .3-3 | High amplitude R waves |
| 4-1-1 . . . . . .4-4 | ST junction (J) and segment depression |
| 5-1 . . . . . .5-4 | T wave items |
| 6-1 . . . . . .6-8 | A-V conduction defect |
| 7-1-1 . . . . . .7-8 | Ventricular conduction defect |
| 8-1-1 . . . . . .8-9 | Arrhythmias |
| 9-1 . . . . . .9-8-2 | Miscellaneous including ST segment elevation (9-2) |
| *Codes 1, 4, 5, and 9-2 (ST elevation) are grouped by leads, resulting in three subclassifications of anterolateral, posterior (inferior) and anterior* | |

**The Minnesota Code Classification System** **for**

**Electrocardiographic Findings**

**Q and QS Patterns**

(Do not code in the presence of WPW code 6-4-1.) To qualify as a Q- or QS-wave, the deflection should be at least 0.1 mV (1 mm in amplitude).

**Anterolateral site (leads I, aVL, V6)**

1-1-1 Q/R amplitude ratio ≥ 1/3, plus Q duration ≥ 0.03 sec in lead I or V6.

1-1-2 Q duration ≥ 0.04 sec in lead I or V6.

1-1-3 Q duration ≥ 0.04 sec, plus R amplitude ≥ 3 mm in lead aVL.

1-2-1 Q/R amplitude ratio ≥ 1/3, plus Q duration ≥ 0.02 sec and < 0.03 sec in lead I or V6.

1-2-2 Q duration ≥ 0.03 sec and < 0.04 sec in lead I or V6.

1-2-3 QS pattern in lead I. Do not code in the presence of 7-1-1.

1-2-8 Initial R amplitude decreasing to 2 mm or less in every beat (and absence of codes 3-2, 7-1-1, 7-2-1, or 7-3 between V5 and V6. (All beats in lead V5 must have an initial R > 2 mm.)

1-3-1 Q/R amplitude ratio ≥ 1/5 and < 1/3, plus Q duration ≥ 0.02 sec and < 0.03 sec in lead I or V6.

1-3-3 Q duration ≥ 0.03 sec and < 0.04 sec, plus R amplitude ≥ 3 mm in lead aVL.

**Posterior (inferior) site (leads II, III, aVF)**

1-1-1 Q/R amplitude ratio ≥ 1/3, plus Q duration ≥ 0.03 sec in lead II.

1-1-2 Q duration ≥ 0.04 sec in lead II.

1-1-4 Q duration ≥ 0.05 sec in lead III, plus a Q-wave amplitude ≥ 1.0 mm in the majority of beats in lead aVF.

1-1-5 Q duration ≥ 0.05 sec in lead aVF.

1-2-1 Q/R amplitude ratio ≥ 1/3, plus Q duration ≥ 0.02 sec and < 0.03 sec in lead II.

1-2-2 Q duration ≥ 0.03 sec and < 0.04 sec in lead II.

1-2-3 QS pattern in lead II. Do not code in the presence of 7-1-1.

1-2-4 Q duration ≥ 0.04 sec and < 0.05 sec in lead III, plus a Q-wave ≥ 1.0 mm amplitude in the majority of beats in aVF.

1-2-5 Q duration ≥ 0.04 sec and < 0.05 sec in lead aVF.

1-2-6 Q amplitude ≥ 5.0 mm in leads III or aVF.

1-3-1 Q/R amplitude ratio ≥ 1/5 and < 1/3, plus Q duration ≥ 0.02 sec and < 0.03 sec in lead II.

1-3-4 Q duration ≥ 0.03 sec and < 0.04 sec in lead III, plus a Q-wave ≥ 1.0 mm amplitude in the majority of beats in lead aVF.

1-3-5 Q duration ≥ 0.03 sec and < 0.04 sec in lead aVF.

1-3-6 QS pattern in each of leads III and aVF. (Do not code in the presence of 7-1-1.)

**Anterior site (leads V1, V2, V3, V4, V5)**

1-1-1 Q/R amplitude ratio ≥ 1/3 plus Q duration ≥ 0.03 sec in any of leads V2, V3, V4, V5.

1-1-2 Q duration ≥ 0.04 sec in any of leads V1, V2, V3, V4, V5.

1-1-6 QS pattern when initial R-wave is present in the adjacent lead to the right on the chest, in any of leads V2, V3, V4, V5, V6.

1-1-7 QS pattern in all of leads V1-V4 or V1-V5.

1-2-1 Q/R amplitude ratio ≥ 1/3, plus Q duration ≥ 0.02 sec and < 0.03 sec, in any of leads V2, V3, V4, V5.

1-2-2 Q duration ≥ 0.03 sec and < 0.04 sec in any of leads V2, V3, V4, V5.

1-2-7 QS pattern in all of leads V1, V2, and V3. (Do not code in the presence of 7-1-1).

1-2-8 Initial R amplitude decreasing to 2.0 mm or less in every beat (and absence of codes 3-2, 7-1-1, 7-2-1, or 7-3) between any of leads V2 and V3, V3 and V4, or V4 and V5. (All beats in the lead immediately to the right on the chest must have an initial R > 2 mm.)

1-3-1 Q/R amplitude ratio ≥ 1/5 and < 1/3 plus Q duration ≥ 0.02 and < 0.03 sec in any of leads V2, V3, V4, V5.

1-3-2 QS pattern in lead V1 and V2. (Do not code in the presence of 3-1 or 7-1-1.)

**QRS Axis Deviation**

(Do not code in presence of low-voltage QRS, code 9-1, WPW 6-4-1, ventricular conduction defects, or 7-1-1, 7-2-1, and 7-4.)

2-1 Left. QRS axis from -300 through -900 in leads I, II, III. (The algebraic sum of major positive and major

negative QRS waves must be zero or positive in I, negative in III, and zero or negative in II.)

2-2 Right. QRS axis from +1200 through -1500 in leads I, II, III. (The algebraic sum of major positive and major

negative QRS waves must be negative in I, and zero or positive in III, and in I must be one-half or more of that in

III.)

2-3 Right (optional code when 2-2 is not present). QRS axis from +900 through +1190 in leads I, II, III. (The algebraic sum of major positive and major negative QRS waves must be zero or negative in I and positive in II and III.)

2-4 Extreme axis deviation (usually S1, S2, S3 pattern). QRS axis from -900 through -1490 in leads I, II, and III (The algebraic sum of major positive and major negative QRS waves must be negative in each of leads I, II, and III.)

2-5 Indeterminate axis QRS axis approximately 900 from the frontal plane. (The algebraic sum of major positive and major negative QRS waves is zero in each of leads I, II and III, or the information from these three leads is incongruous.)

**High Amplitude R Waves**

3-1 Left: R amplitude > 26 mm in either V5 or V6, or R amplitude > 20.0 mm in any of leads I, II, III, aVF, or R amplitude > 12.0 mm in lead aVL. (All criteria measured only on second to last complete normal beat.)

3-2 Right: R amplitude ≥ 5.0 mm and R amplitude ≥ S amplitude in the majority of beats in lead V1, when S amplitude is > R amplitude somewhere to the left on the chest of V1 (codes 7-3 and 3-2, if criteria for both are present).

3-3 Left (optional code when 3-1 is not present): R amplitude > 15.0 mm but " 20.0 mm in lead I, or R amplitude in V5 or V6, plus S amplitude in V1 > 35.0 mm. (Measured only on second to last complete normal beat.)

3-4 Criteria for 3-1 and 3-2 both present.

**ST Junction (J) and Segment Depression**

(Do not code in the presence of codes 6-4-1, 7-1-1, 7-2-1 or 7-4. When 4-1, 4-2, or 4-3 is coded, then a 5-code must also be assigned except in lead V1.)

**Anterolateral site (leads I, aVL, V6)**

4-1-1 STJ depression ≥ 2.0 mm and ST segment horizontal or downward sloping in any of leads I, aVL, or V6.

4-1-2 STJ depression ≥ 1.0 mm but < 2.0 mm, and ST segment horizontal or downward sloping in any of leads I,aVL, or V6.

4-2 STJ depression ≥ 0.5 mm and < 1.0 mm and ST segment horizontal or downward sloping in any of leads I, aVL, or V6.

4-3 No STJ depression as much as 0.5 mm but ST segment downward sloping and segment or T-wave nadir ≥ 0.5 mm below P-R baseline, in any of leads I, aVL, or V6.

4-4 STJ depression ≥ 1.0 mm and ST segment upward sloping or U-shaped, in any of leads I, aVL, or V6.

**Posterior (inferior) site (leads II, III, aVF)**

4-1-1 STJ depression ≥ 2.0 mm and ST segment horizontal or downward sloping in lead II or aVF.

4-1-2 STJ depression ≥ 1.0 mm but < 2.0 mm and ST segment horizontal or downward sloping in lead II or aVF.

4-2 STJ depression ≥ 0.5 mm and < 1.0 mm and ST segment horizontal or downward sloping in lead II or aVF.

4-3 No STJ depression as much as 0.5 mm, but ST segment downward sloping and segment or T-wave nadir ≥ 0.5 mm below P-R baseline in lead II.

4-4 STJ depression ≥ 1.0 mm and ST segment upward sloping, or U-shaped, in lead II.

**Anterior site (leads V1, V2, V3, V4, V5)**

4-1-1 STJ depression ≥ 2.0 and ST segment horizontal or downward sloping in any of leads V1, V2, V3, V4, V5.

4-1-2 STJ depression ≥ 1.0 mm but < 2.0 mm and ST segment horizontal or downward sloping in any of leads V1, V2, V3, V4, V5

4-2 STJ depression ≥ 0.5 mm and < 1.0 mm and ST segment horizontal or downward sloping in any of leads V1, V2, V3, V4, V5.

4-2 No STJ depression as much as 0.5 mm, but ST segment downward sloping and segment or

T-wave nadir ≥ 0.5 mm below P-R baseline in any of leads V2, V3, V4, V5.

4-4 STJ depression ≥ 1.0 mm and ST segment upward sloping or U-shaped in any of leads V1, V2, V3, V4, V5.

**T-Wave Items**

(Do not code in the presence of code 6-4-1, 7-1-1, 7-2-1 or 7-4.)

**Anterolateral site (leads I, aVL, V6)**

5-1 T amplitude negative 5.0 mm or more in either of leads I, V6, or in lead aVL when R amplitude is ≥ 5.0 mm.

5-2 T amplitude negative or diphasic (positive-negative or negative-positive type) with negative phase at least 1.0

mm but not as deep as 5.0 mm in lead I or V6, or in lead aVL when R amplitude is ≥ 5.0 mm.

5-3 T amplitude zero (flat), or negative, or diphasic (negative-positive type only) with less than 1.0 mm negative phase in lead I or V6, or in lead aVL when R amplitude is ≥ 5.0 mm.

5-4 T amplitude positive and T/R amplitude ratio < 1/20 in any of leads I, aVL, V6; R wave amplitude must be ≥10.0 mm.

**Posterior (inferior) site (leads II, III, aVF)**

5-1 T amplitude negative 5.0 mm or more in lead II, or in lead aVF when QRS is mainly upright.

5-2 T amplitude negative or diphasic with negative phase (negative-positive or positive-negative type) at least 1.0 mm but not as deep as 5.0 mm in lead II, or in lead aVF when QRS is mainly upright.

5-3 T amplitude zero (flat), or negative, or diphasic (negative-positive type only) with less than 1.0 mm negative phase in lead II; not coded in lead aVF.

5-4 T amplitude positive and T/R amplitude ratio < 1/20 in lead II; R wave amplitude must be ≥ 10.0 mm.

**Anterior site (leads V2, V3, V4, V5)**

5-1 T amplitude negative 5.0 mm or more in any of leads V2, V3, V4, V5.

5-2 T amplitude negative (flat), or diphasic (negative-positive or positive-negative type) with negative phase at least 1.0 mm but not as deep as 5.0 mm, in any of leads V2, V3, V4, V5.

5-3 T amplitude zero (flat), or negative, or diphasic (negative-positive type only) with less than 1.0 mm negative phase, in any of leads V3, V4, V5.

5-4 T amplitude positive and T/R amplitude ratio < 1/20 in any of leads V3, V4, V5; R wave amplitude must be≥10.0 mm.

**A-V Conduction Defect**

6-1 Complete (third degree) A-V block (permanent or intermittent) in any lead. Atrial and ventricular complexes independent, and atrial rate faster than ventricular rate, with ventricular rate < 60.

6-2-1 Mobitz Type II (occurrence of P-wave on time with dropped QRS and T).

6-2-2 Partial (second degree) A-V block in any lead (2:1 or 3:1 block).

6-2-3 Wenckebach's Phenomenon (P-R interval increasing from beat to beat until QRS and T dropped).

6-3 P-R (P-Q) interval ≥ 0.22 sec in the majority of beats in any of leads I, II, III, aVL, aVF.

6-4-1 Wolff-Parkinson-White Pattern (WPW), persistent. Sinus P-wave. P-R interval < 0.12 sec, plus QRS duration ≥ 0.12 sec, plus R peak duration ≥ 0.06 sec, coexisting in the same beat and present in the majority of beats in any of leads I, II, aVL, V4, V5, V6. (6-4-1 suppresses 1-2-3, 1-2-7, 1-2-8, 1-3-2, 1-3-6, all 3, 4, 5, 9-2, 9-4, 9-5 codes.)

6-4-2 WPW Pattern, intermittent. WPW pattern in " 50% of beats in appropriate leads.

6-5 Short P-R interval. P-R interval < 0.12 sec in all beats of any two of leads I, II, III, aVL, aVF.

6-6 Intermittent aberrant atrioventricular conduction. P-R > 0.12 sec (except in presence of 6-5 or heart rate greater than 100); wide QRS complex > 0.12 sec; normal P-wave when most beats are sinus rhythm. (Do not code in the presence of 6-4-2.)

6-8 Artificial pacemaker.

**Ventricular Conduction Defect**

7-1-1 Complete left bundle branch block (LBBB). (Do not code in presence of 6-1, 6-4-1, 6-8, 8-2-1 or 8-2-2.) QRS duration ≥ 0.12 sec in a majority of beats in any of leads I, II, III, aVL, aVF, *plus* R peak duration ≥ 0.06 sec in a majority of beats (of the same QRS pattern) in any of leads I, II, aVL, V5, V6. (7-1-1 suppresses 1-2-3, 1-2-7, 1-2-8, 1-3-2, 1-3-6, all 2, 3, 4, 5, 9-2, 9-4, 9-5 codes. If any other codable Q-wave coexists with the LBBB pattern, code the Q and diminish the 7-1-1 code to a 7-4 code.)

7-1-2 Intermittent left bundle branch block. Same as 7-1-1 but with presence of normally conducted QRS complexes of different shape than the LBBB pattern.

7-2-1 Complete right bundle branch block (RBBB). (Do not code in the presence of 6-1, 6-4-1, 6-8, 8-2-1 or 8-2-2.) QRS duration ≥ 0.12 sec in a majority of beats in any of leads I, II, III, aVL, aVF, *plus*: R' > R in V1 or V2; or QRS mainly upright, with R peak duration ≥ 0.06 sec in V1 or V2; or S duration >R duration in all beats in lead I or II. (7-1 suppresses 1-2-3, 1-2-7, 1-2-8, 1-3-2, 1-3-6, all 2, 3, 4, 5, 9-2, 9-4, 9-5 codes.

7-2-2 Intermittent right bundle branch block. Same as 7-2-1 but with presence of normally conducted QRS complexes of different shape than the RBBB pattern.

7-3 Incomplete right bundle branch block. QRS duration < 0.12 sec in each of leads I, II, III, aVL, aVF, and R' > R in either of leads V1, V2.(Code as 3-2 in addition if those criteria are met. 7-3 suppresses code 1-2-8.)

7-4 Intraventricular block. QRS duration ≥ 0.12 sec in a majority of beats in any of leads I, II, III, aVL, aVF. (7-4 suppresses all 2, 3, 4, 5, 9-2, 9-4, 9-5 codes.)

7-5 R-R' pattern in either of leads V1, V2 with R' amplitude ≥ R.

7-6 Incomplete left bundle branch block. (Do not code in the presence of any codable Q- or QS-wave.) QRS duration ≥ 0.10 sec and < 0.12 in the majority of beats of each of leads I, aVL, and V5 or V6.

7-7 Left anterior hemiblock (LAH). QRS duration < 0.12 sec in the majority of beats in leads I, II, III, aVL, aVF, plus Q-wave amplitude ≥ 0.25 mm and < 0.03 sec duration in lead I, plus left axis deviation of -450 or more negative. (In presence of 7-2, code 7-8 if axis is < -450 and the Q-wave in lead I meets the above criteria.)

7-8 Combination of 7-7 and 7-2.

**Arrhythmias**

8-1-1 Presence of frequent atrial or junctional premature beats (10% or more of recorded complexes).

8-1-2 Presence of frequent ventricular premature beats (10% or more of record complexes).

8-1-3 Presence of both atrial and/or junctional premature beats and ventricular premature beats (so that individual frequencies are < 10% but *combined* premature beats are ≥ 10% of complexes).

8-1-4 Wandering atrial pacemaker.

8-1-5 Presence of 8-1-2 and 8-1-4.

8-2-1 Ventricular fibrillation or ventricular asystole.

8-2-2 Persistent ventricular (idioventricular) rhythm.

8-2-3 Intermittent ventricular tachycardia. Three or more consecutive ventricular premature beats occurring at a rate ≥ 100. This includes more persistent ventricular tachycardia.

8-2-4 Ventricular parasystole (should not be coded in presence of 8-3-1).

8-3-1 Atrial fibrillation (persistent).

8-3-2 Atrial flutter (persistent).

8-3-3 Intermittent atrial fibrillation (code if 3 or more clear-cut, consecutive sinus beats are present in any lead).

8-3-4 Intermittent atrial flutter (code of 3 or more clear-cut, consecutive sinus beats are present in any lead).

8-4-1 Supraventricular rhythm persistent. QRS duration < 0.12 sec; and absent P-waves or presence of abnormal Pwaves (inverted or flat in aVF); and regular rhythm.

8-4-2 Supraventricular tachycardia intermittent. Three consecutive atrial or junctional premature beats occurring at a rate ≥ 100.

8-5-1 Sinoatrial arrest. Unexpected absence of P, QRS and T, plus a R-R interval at a fixed multiple of the normal interval, ± 10%.

8-5-2 Sinoatrial block. Unexpected absence of P, QRS and T, preceded by progressive shortening of P-P intervals. (RR interval at a fixed multiple of the normal interval, ± 10%.

8-6-1 A-V dissociation with ventricular pacemaker (without capture). Requires: P-P and R-R occur at variable rates with ventricular rate as fast as or faster than the atrial rate, plus variable P-R intervals, plus no capture beats.

8-6-2 A-V dissociation with ventricular pacemaker (with capture).

8-6-3 A-V dissociation with atrial pacemaker (without capture).

8-6-4 A-V dissociation with atrial pacemaker (with capture).

8-7 Sinus tachycardia (over 100/min).

8-8 Sinus bradycardia (under 50/min).

8-9 Other arrhythmias. Heart rate may be recorded as a continuous variable.

**ST Segment Elevation**

**Anterolateral site (leads I, aVL, V6)**

9-2 ST segment elevation ≥ 1.0 mm in any of leads I, aVL, V6.

**Posterior (inferior) site (leads II, III, aVF)**

9-2 ST segment elevation ≥ 1.0 mm in any of leads II, III, aVF.

**Anterior site (leads V1, V2, V3, V4, V5)**

9-2 ST segment elevation ≥ 1.0 mm in lead V5 or ST segment elevation ≥2.0 mm in any of leads V1, V2, V3, V4.

**Miscellaneous Items**

9-1 Low QRS amplitude. QRS peak-to-peak amplitude < 5 mm in all beats in each of leads I, II, III, or < 10 mm in all beats in each of leads V1, V2, V3, V4, V5, V6. (Check calibration before coding.)

9-3 P-wave amplitude ≥ 2.5 mm in any of leads II, III, aVF, in a majority of beats.

9-4-1 QRS transition zone at V3 or to the right of V3 on the chest. (Do not code in the presence of 6-4-1, 7-1-1, 7-2-1 or 7-4.)

9-4-2 QRS transition zone at V4 or to the left of V4 on the chest. (Do not code in the presence of 6-4-1, 7-1-1, 7-2-1 or 7-4.)

9-5 T-wave amplitude > 12 mm in any of leads I, II, III, aVL, aVF, V1, V2, V3, V4, V5, V6. (Do not code in the presence of 6-4-1, 7-1-1, 7-2-1 or 7-4.)

9-8-1 Technical problems which interfere with coding.

9-8-2 Technical problems which do not interfere with coding.
